# Routine Forearm Cast Splitting in Pediatric Patients Following Closed Reduction

**DOI:** 10.7759/cureus.116309

**Published:** 2026-09-15

**Authors:** Lavell Manuopangai, Jihae Abou El Ela, Viren Kasipersad

**Affiliations:** 1 Orthopaedic Surgery, Rotorua Hospital, Rotorua, NZL

**Keywords:** cast-related complications, cast splitting, fracture displacement, loss of reduction, pediatric forearm fractures

## Abstract

Introduction

Routine cast splitting following closed reduction of pediatric forearm fractures is commonly performed to accommodate swelling and reduce cast-related complications. However, evidence supporting routine splitting remains conflicting. This study aimed to compare cast-related complications between split and non-split casts and assess the associations of cast splitting, fracture displacement, and gap index with clinical outcomes.

Materials and methods

A retrospective cohort study was performed of children aged two to 16 years with forearm fractures initially managed with cast immobilization between January 2022 and December 2025. Demographic, clinical, and radiographic data were collected, including cast-splitting status, indication for splitting, fracture displacement, gap index, cast-related complications, and subsequent operative management. The gap index was categorized as ≤0.15 or >0.15. Results were summarized using frequencies and proportions for categorical variables and logistic regression to examine outcomes of interest.

Results

A total of 146 patients met the inclusion criteria, of whom 42 (28.8%) underwent cast splitting and 104 (71.2%) did not. Of the 42 split casts, 39 (92.9%) were split routinely. Cast-related complications were uncommon. Three patients (2.9%) initially managed without splitting returned for delayed cast splitting, and one patient with a wet cast needed a cast change, while one patient (2.4%) in the split group returned with a broken cast requiring a cast change. No cases of compartment syndrome occurred. Gap index ≤0.15 was not associated with cast-splitting status (p=0.30), and no significant association was identified between the gap index category and cast-related complications. Fifteen patients subsequently required operative management following loss of reduction. Operative management was associated with cast splitting (p=0.033) and initial fracture displacement (p=0.001).

Conclusions

Cast-related complications were uncommon irrespective of cast-splitting practice. These findings suggest that selective splitting based on clinical assessment and risk factors may be appropriate. Gap index was not associated with cast complications. Cast splitting was associated with subsequent operative management; however, this association should be interpreted cautiously given the retrospective nature of the study and other important variables not assessed in this cohort.

## Introduction

Forearm fractures are among the most common pediatric fractures [1] and are frequently managed with closed reduction and cast immobilization. A well-molded cast remains central to non-operative treatment [1].

Routine cast splitting after reduction is commonly performed in many centers, including our institution. This is historically believed to accommodate swelling and reduce the risk of pain, neurovascular compromise, or other cast-related complications. Udall et al. [2] support this practice. There are conflicting results in the literature regarding routine splitting of forearm casts in the pediatric population. Randomized controlled trials have shown no significant difference in complication rates comparing circumferential and split casts [3, 4]. On the other hand, experimental studies have shown the benefit of splitting casts [5, 6]. Cast splitting may be appropriate in the setting of a clinically tight cast, marked swelling, or neurovascular concern [3]. Routine splitting also has potential disadvantages, including cast weakening, loss of cast molding, and cast saw injuries [3]. It also limits the ability to wedge the cast if needed during early follow-up. It remains unclear whether routine splitting is necessary in all children following reduction of a forearm fracture [3, 7] or whether a more selective approach may be sufficient.

The cast index has been well-studied [8], and a value of <0.775 has been shown to be associated with tight cast syndrome [9]. The gap index [10] is an indicator of cast molding and padding [11]. The gap index reflects cast conformity and the amount of padding [11] between the limb and the cast.

The aims of this study were to (1) compare rates of cast-related complications between routine cast splitting and no cast splitting; (2) assess if cast splitting and/or fracture displacement results in loss of reduction; and (3) calculate if the gap index has any association with cast-related complications.

## Materials and methods

The study was conducted at a regional hospital in the mid-North Island in New Zealand. The regional hospital also serves two smaller rural hospitals. The majority of pediatric forearm fractures undergo assessment, closed reduction, and casting by emergency physicians. Standard practice is to use Soffban® (Smith and Nephew, Mölndal, Sweden) to protect the skin and a plaster of Paris cast in the acute setting. Orthopedic referral is only made when there are concerns regarding inadequate closed reduction or if there are suitable indications for operative management.

Study design

A retrospective cohort study was performed at Rotorua Hospital, Rotorua, New Zealand. Ethics approval was obtained from the Lakes Research and Ethics Committee, Health New Zealand Te Whatu Ora Lakes, on 17 July 2026. The committee considered the study scientifically and ethically sound. Given the retrospective nature of the study and that no patient identification was used, consent was waived. Electronic medical records of pediatric patients treated for forearm fractures with cast immobilization from January 2022 to December 2025 were identified.

Participants

Children aged two to 16 years with forearm fractures (metaphyseal, diaphyseal, both bone fractures, and Salter-Harris fracture types) who were initially treated with plaster cast immobilization were eligible for inclusion. Fractures were classified as either displaced or non-displaced. Fractures were considered displaced if there was at least 5 mm displacement or angulation greater than 10 degrees in either plane. Patients were included if adequate post-reduction radiographs were available to allow measurement of the gap index. Patients were excluded if they had incomplete radiographic or clinical records, if their fracture was not managed with cast immobilization or complex fractures, if they underwent immediate operative fixation at the index presentation, or if their follow-up was undertaken at another institution.

Data collection

National hospital numbers were generated from discharge coding criteria using the Australian Modification of the World Health Organization International Statistical Classification of Diseases and Related Health Problems, 10^th^ Revision system (ICD-10-AM) [12]. Electronic records and radiographs were reviewed using the regional Picture Archiving and Communication System (PACS) and hospital clinical records portal. The following variables were collected: age at injury, sex, fracture type, cast split status at index treatment, indication for cast split, gap index, cast-related complications, and delayed operative management. Cast-related complications and unplanned return to hospital were analyzed from the day of discharge to initial orthopedic follow-up, which was usually seven to 10 days later. Casts were usually modified during the first follow-up, and the scope of this study was to capture short-term cast complications related to initial cast splitting. Decisions for operative management were also made at the first follow-up. Two orthopedic registrars performed electronic chart reviews independently, and an orthopedic consultant reconciled any discrepancies.

Radiographic measurements

Gap Index

Malviya et al. [11] described the calculation of the gap index as follows: calculated from available plain radiographs by measuring the gap distance between the skin and inner cast on the radial and ulnar sides of the anteroposterior (AP) radiograph. These values were added together and then divided by the AP inner diameter of the cast. On the lateral radiograph, the gap distance between the skin and cast was measured on the volar and dorsal sides. These values were added together and divided by the inner cast diameter. The AP and lateral ratios were summed to calculate the gap index [10]. Values of ≤ 0.15 indicate adequate cast conformity. [10]

Outcome measures

The outcome measures were cast-related complications and secondary loss of reduction leading to operative management in the two groups (cast split vs. no cast split).

Statistical analysis

The results were summarized using frequencies and proportions for categorical variables. Logistic regression was carried out to examine the risk factors and outcome of interest. Logistic regression comparing operative or not and cast split was performed after adjusting for fracture type. Inter-rater agreement was examined using Kappa (κ) statistics and intraclass correlation coefficient (ICC). Statistical analyses were carried out using SAS 9.4 (SAS Institute Inc., Cary, NC, USA). The gap index was categorized as ≤0.15 or >0.15, and its association with cast-splitting status and cast-related complications was assessed using Fisher’s exact or chi-square testing, as appropriate. Given the retrospective nature of the study, it was deemed not necessary to calculate sample size and power.

## Results

A total of 337 patients were identified from the initial search using discharge coding criteria. Of these, the following patients were excluded: 71 patients outside the study time period, 11 duplicates, 95 patients who were treated operatively at the time of first admission, and 14 patients who were lost to follow-up in other regions. Therefore, 146 patients met the inclusion criteria and were included in the study. This is represented in Figure 1.

**Figure 1:**
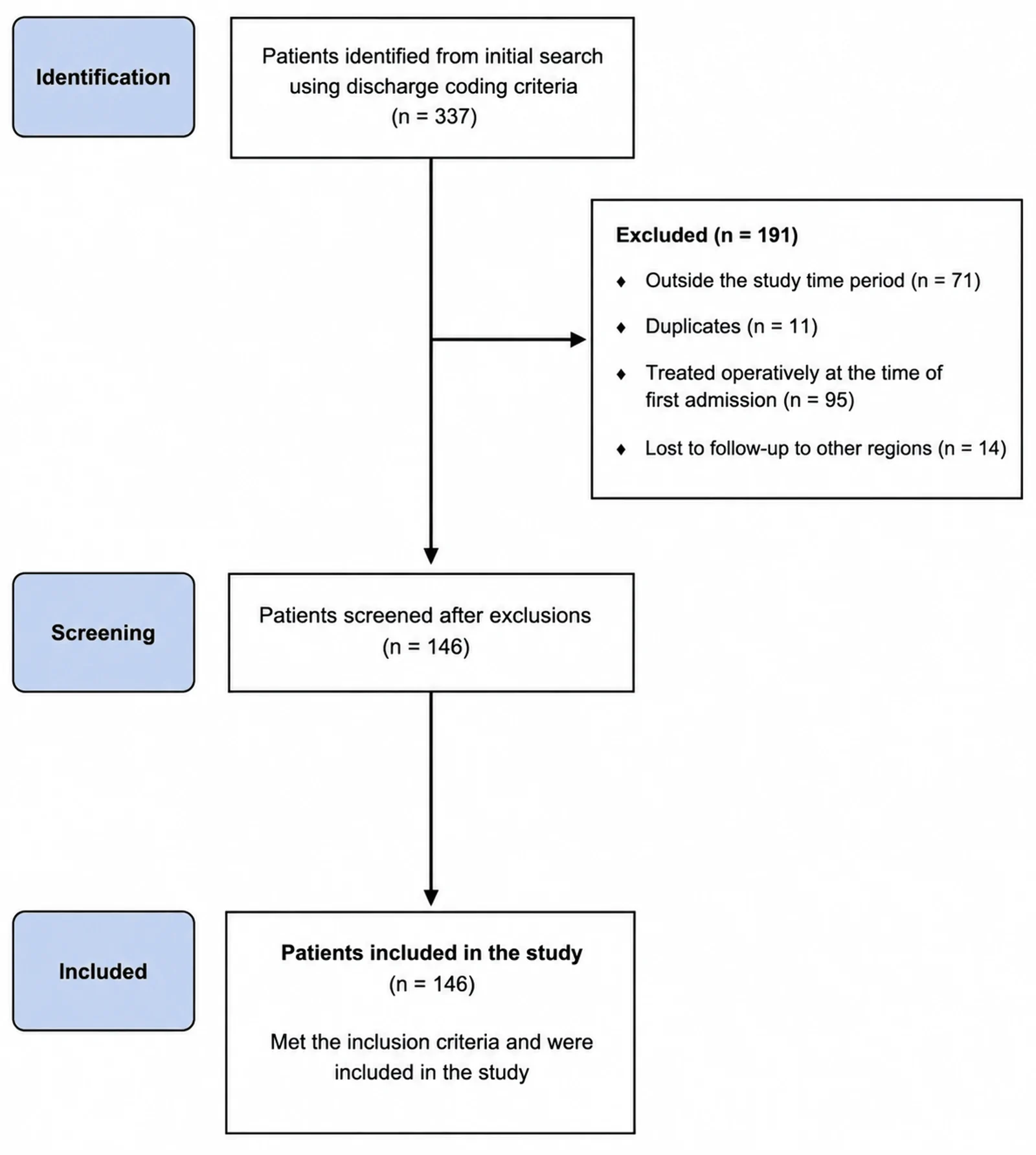
Flow diagram of the patient selection process

Table 1 shows the comparisons of the cast-split group and the no-cast-split group. It shows that 42 patients underwent cast splitting and 104 did not. Of the 42 patients who underwent cast splitting, 39 (92.9%) were split routinely and three were split for clinical concerns, as also shown in Table 1. The casts that were split due to clinical concerns were done by the treating doctor at the time the cast was applied and documented in the clinical notes prior to discharge. Cast-related complications were uncommon, with three patients (2.0%) initially managed without cast splitting, subsequently returning for delayed cast splitting; one patient (0.96%) returned with a wet cast; and only one patient (2.4%) in the cast split group returned with a broken cast requiring a cast change. No cases of compartment syndrome were seen. Table 1 shows that a gap index of ≤0.15 was identified in 13.6% of patients and was not associated with cast splitting status (p=0.30). No significant association was observed between gap index category and cast-related complications.

**Table 1:** Comparison of cast-split and non-split groups X^2^: chi square test

			Cast-Split Group or Non-split Group		
Outcomes		Total n(%)	Yes n(%)	No n(%)	p-value
		146 (100)	42 (28.76)	104 (71.23)	
Indications for Cast Split	Routine		39 (92.87)		
	Compound		1 (2.38)		
	Numbness		1 (2.38)		
	Swelling		1 (2.38)		
Gap index	≤ 0.15	20 (13.61)	7 (16.28)	13 (12.5)	0.30 by X^2 ^test
	> 0.15	126 (86.39)	35 (83.72)	91 (87.5)	

Table 2 compares patients who successfully completed non-operative management with cast treatment with those who experienced loss of reduction at first follow-up and required operative management. It shows that 15 patients underwent conversion to operative management after the failure of non-operative management due to loss of reduction at the first follow-up visit. One hundred and thirty-one patients were successfully managed non-operatively in a cast. Patients who required surgery were more likely to have undergone cast splitting (p = 0.033), although they also had a significantly greater proportion of displaced fractures (p = 0.001), confounding this finding as shown in Table 2. Routine cast splitting was also more common among patients who subsequently required operative intervention. Fracture displacement was strongly associated with operative management. Displaced fractures accounted for 60% of operative cases compared with 19.08% of non-operative cases (p = 0.001). Non-displaced fractures were more frequently managed successfully without surgery. 

**Table 2:** Comparison of non-operative and operative (loss of reduction) groups

			Group			
Characteristics		Total N (%)	Operative N (%)	Non-operative N (%)	OR (95CI)	p-value
	Total	146 (100)	15 (10.27)	131 (89.72)		
Demographic Characteristics						
Gender	Female	49 (33.56)	2 (13.33)	47 (35.88)	0.28 (0.06 – 1.27)	0.098
	Male	97 (66.44)	13 (86.67)	84 (64.12)	1	
Age (Years)	Mean (SD)	8.45 (3.52)	10.1 (4.40)	8.26 (3.38)	1.15 (0.98 – 1.35)	0.09
Clinical Characteristics						
Cast Split	Yes	42 (28.77)	8 (53.33)	34 (25.95)	3.26 (1.10 – 9,67)	0.033
	No	104 (71.23)	7 (46.67)	97 (74.05)	1	
Fracture Type	Non- displaced	112 (76.71)	6 (40)	106 (80.92)	1	0.001
	Displaced	34 (23.29)	9 (60)	25 (19.08)	6.36 (2.08 – 19.5)	

Table 3 shows adjustment analysis for fracture type in the cast split operative group. Cast splitting was significantly associated with operative treatment. Eight of 42 patients (19.0%) who underwent cast splitting required operative management, compared with seven of 104 patients (6.7%) without cast splitting. The unadjusted odds of operative treatment were significantly higher following cast splitting (OR 3.26, 95% CI 1.10-9.67; p=0.033). After adjustment for fracture type, cast splitting remained independently associated with operative treatment (adjusted OR 5.32, 95% CI 1.52-18.7; p=0.009).

**Table 3:** Adjustment analysis of cast split in the operative group

			Group					
		Total N(%)	Operative N(%)	Non-operative N(%)	OR (95 CI)	p-value	OR (95 CI)	p-value
Cast Split	Yes	42 (28.77)	8 (53.33)	34 (25.95)	3.26 (1.10 – 9,67)	0.033	5.32 (1.52 – 18.7)	0.009
	No	104 (71.23)	7 (46.67)	97 (74.05)	1		1	

Inter-rater reliability was excellent for operative status (κ=0.93, 95% CI 0.82-1.00) and cast splitting (κ=0.92, 95% CI 0.85-0.99). Agreement for fracture type was moderate (κ=0.52, 95% CI 0.35-0.70). Gap index measurements demonstrated moderate-to-good inter-rater reliability (ICC=0.67, 95% CI 0.56-0.75). This is demonstrated in Table 4. 

**Table 4:** Agreement between the two raters ICC: intraclass correlation coefficient

Operative					
		Rater1			
Rater2		Acute	Non-operative		
	Acute	14 (93.33)	1 (0.76)	Kappa (95% CI)	p
	Non-operative	1 (6.67)	130 (99.24)	0.93 (0.82 - 1.00)	1
Fracture Type					
		Rater1			
Rater2		Non displaced	Displaced	Kappa (95% CI)	
	Non-displaced	109 (97.32)	18 (52.94)	0.52 (0.35 - 0.70)	0.0011
	Displaced	3 (2.68)	16 (47.06)		
Cast Split					
		Rater1			
Rater2		Yes	No	Kappa (95% CI)	
	Yes	40 (95.24)	3 (2.88)	0.92 (0.85 - 0.99)	0.6547
	No	2 (4.76)	101 (97.12)		
Gap Index: Rater 1 vs. Rater 2					
				ICC (95% CI)	
				0.67 (0.56 - 0.75)	2.42 x 10^-21^

Among the 15 patients who required operative management following loss of reduction, 10 (66.7%) had diaphyseal fractures, and five (33.3%) had metaphyseal fractures. The operative cohort was predominantly male (13/15, 86.7%), with ages ranging from two to 14 years. Loss of reduction varied in severity and included angulation of 10°-40°, partial displacement of 25%-50%, and complete (100%) displacement. Several cases demonstrated substantial displacement or angulation at the time loss of reduction was identified

## Discussion

This study demonstrated low cast-related complication rates of 3.85% in the non-split group compared with 2.38% in the split group. Owing to the small number of complication events, statistical testing for individual complications was not considered reliable. Regardless, the low complication rate is consistent with retrospective studies reporting low rates of cast-related complications and emergency department revisits for cast-related pain [12, 13]. This is also supported by randomized controlled trials showing no significant difference in cast-related complications whether casts were bivalved or univalved compared with intact casts [3, 4]. In our cohort, five patients experienced cast complications. In the non-split group, this consisted of three patients (2.0%) initially treated without cast splitting who were re-presented and required cast splitting, and one patient returned with a wet cast. One patient returned with a broken cast in the cast-split group. Of the patients returning for cast split, two returned with increased swelling of the hand, and one patient returned with increased pain. All three patients had displaced fractures, indicating an increased risk of swelling related to the initial injury and closed reduction [9]. These displaced fractures may indicate a higher-energy mechanism and more soft tissue oedema [9]. A retrospective study reported that patients with displaced fractures and associated distal ulna fracture are at greater risk of tight cast syndrome and should be followed up closely [9]. Our study showed that a total of 112 patients had non-displaced fractures. No patients in this group experienced cast-related complications requiring delayed splitting. These results from our cohort suggest that selective cast splitting may be more appropriate following reduction in patients who are at risk of tight cast syndrome. Reported risk factors from other studies include a cast index of <0.775 [9] and patients in a setting of polytrauma [14]. In contrast, patients with low-energy trauma [3] and non-displaced fractures could be treated with circumferential casts without splitting. Counselling at-risk patients and families with return instructions can be appropriate following reduction and casting.

Our cohort showed that patients who underwent operative management after secondary loss of reduction at follow-up were more likely to have had their casts split (8/15, 53%), which raises the question of whether cast splitting is a significant factor. Unadjusted, cast splitting was statistically associated with subsequent operative management. Eight out of 42 (19%) patients who underwent cast splitting required operative management compared with seven out of 104 patients (6.7%) without cast splitting. This was confounded by the fact that these patients were also more likely to present with displaced fractures (9/15, 60%). This may reflect greater initial fracture displacement rather than an independent effect of cast splitting. Fracture displacement appeared to be a stronger predictor of treatment failure in our cohort, with p = 0.001. Cast splitting could also be a surrogate marker for clinician concern regarding fracture displacement, as clinicians may preferentially split casts for more displaced fractures, so this association may be confounded. This is supported by a retrospective study showing that initial displacement is a major risk factor for loss of reduction [15]. One of the randomized controlled trials comparing the two groups showed greater secondary displacement of the fracture in the split-cast group; however, this did not reach statistical significance. Interestingly, when we adjusted for displacement by performing multivariate logistic regression testing to separate their effects, cast splitting still remained independently associated with operative management (p = 0.009). Operative management is 5.3 times more likely to occur in patients with cast split compared with no cast split after adjusting for fracture type. Multivariable logistic regression demonstrated that cast splitting remained significantly associated with loss of reduction after adjusting for initial fracture displacement, suggesting that the observed association was not solely explained by differences in fracture displacement between the groups. However, loss of reduction is multifactorial, and other important factors, such as fracture pattern, degree of initial displacement, quality of reduction, and other radiographic measures of cast quality, were not fully accounted for in this present study. Therefore, residual confounding from unmeasured variables cannot be excluded, and the observed association between cast splitting and loss of reduction should be interpreted very cautiously.

The gap index has primarily been studied in relation to loss of reduction and fracture re-displacement [16]. Gap index measurements were not associated with cast-splitting practice in our cohort. The absence of increased cast-related complications in patients with lower gap indices suggests that it may not reliably reflect clinically significant cast tightness. Although the results from our cohort did not show a statistically significant difference, our sample size was possibly not large enough to detect a significant difference and may be underpowered. Unlike surface-pressure testing described in the randomized controlled trial, the gap index has not been validated for predicting cast-related complications [5] and may not reflect clinically significant constriction. The cast index was not calculated in the present study. However, it is a well-established radiographic measure of a predictor of loss of reduction [8]; therefore, the appropriate cast application in maintaining fracture stability is important.

The main strength of this study is the independent dual-reviewer data extraction with consultant adjudication. This was assessed using the inter-rater reliability, which we found was excellent for operative status and cast splitting (κ=0.93 and κ=0.92, respectively), moderate for fracture type, and moderate to good for gap index measurements (κ=0.52 and κ=0.67, respectively).

Limitations of this study

The retrospective nature of this study is susceptible to selection bias. There is a risk of allocation bias because the clinician’s decision to split the cast may have been influenced by factors associated with the risk of complications. Patients who had their cast split were confounded by the fact that they were also more likely to have displaced fractures, therefore leading to clinician concerns. This study is a single-center study in a regional hospital, limiting the generalizability of the findings. The small number of complication events limits statistical power, and the true difference between groups may not be detected. Patients were counselled on discharge to return if any concerns. Although hospital records were available for the region, there remains a possibility that patients may have sought care with their general practitioner or community-based urgent care clinics. Due to inconsistency of documentation, we were not able to subgroup the cast split group into cast length, bivalve, or univalve splits, as the type of split was not clearly documented, which would have been clinically useful. Some casts were also not documented to have a split but were only noted on post-cast X-rays. The cast index was not calculated in our cohort, which prevented direct assessment of the influence of cast molding quality on maintenance of fracture reduction. The cast-related complications after the first follow-up were not analyzed. The two orthopedic registrars who collected and analyzed data were not blinded to the measured outcomes. Finally, discharge coding is dependent on clinician record documentation. If diagnoses at discharge were inaccurate or incomplete, the coding criteria may not have captured all patients who would have been eligible for inclusion in this study.

## Conclusions

In our cohort, cast splitting was associated with loss of reduction requiring operative management after adjustment for fracture displacement; however, unmeasured factors may also contribute to this association, and causality cannot be fully established. This should be interpreted cautiously given the retrospective nature of this study. The gap index was not significantly associated with cast complications; however, the small number of events limits definitive conclusions regarding its predictive value. Cast-related complications were uncommon in this cohort. These findings do not support the routine splitting of all casts following closed reduction of pediatric forearm fractures and suggest that a more selective approach based on clinical assessment and risk factors may be appropriate.
